# Hyperspectral imaging in systemic sclerosis-associated Raynaud phenomenon

**DOI:** 10.1186/s13075-023-02990-3

**Published:** 2023-01-20

**Authors:** Shannon Teaw, Akash Gupta, Alyssa Williams, F. Perry Wilson, Brandon J. Sumpio, Bauer E. Sumpio, Monique Hinchcliff

**Affiliations:** 1 Section of Rheumatology, Allergy & Immunology, Department of Medicine, Yale School of Medicine, 300 Cedar Street, The Anlyan Center PO Box 208031, New Haven, CT 06520 USA; 2 Clinical and Translational Research Accelerator, Department of Medicine, Yale School of Medicine, New Haven, CT USA; 3 Department of Internal Medicine, Section of Nephrology, Yale School of Medicine, New Haven, CT USA; 4 Division of Vascular and Endovascular Surgery, Massachusetts General Hospital, Boston, MA USA; 5 Division of Vascular Surgery, Department of Surgery, Yale School of Medicine, New Haven, CT USA

**Keywords:** Systemic sclerosis, Scleroderma, Hyperspectral imaging, Raynaud phenomenon, Patient-reported outcome instruments, Imaging, Outcomes, Digital ulcers, Raynaud condition score, Cochin hand function scale

## Abstract

**Background/purpose:**

Lack of robust, feasible, and quantitative outcomes impedes Raynaud phenomenon (RP) clinical trials in systemic sclerosis (SSc) patients. Hyperspectral imaging (HSI) non-invasively measures oxygenated and deoxygenated hemoglobin (oxyHb and deoxyHb) concentrations and oxygen saturation (O_2_ sat) in the skin and depicts data as oxygenation heatmaps. This study explored the potential role of HSI in quantifying SSc-RP disease severity and activity.

**Methods:**

Patients with SSc-RP (*n* = 13) and healthy control participants (HC; *n* = 12) were prospectively recruited in the clinic setting. Using a hand-held camera, bilateral hand HSI (HyperMed™, Waltham, MA) was performed in a temperature-controlled room (22 °C). OxyHb, deoxyHb, and O_2_ sat values were calculated for 78-mm^2^ regions of interest for the ventral fingertips and palm (for normalization). Subjects underwent a cold provocation challenge (gloved hand submersion in 15 °C water bath for 1 min), and repeated HSI was performed at 0, 10, and 20 min. Patients completed two patient-reported outcome (PRO) instruments: the Raynaud Condition Score (RCS) and the Cochin Hand Function Scale (CHFS) for symptom burden assessment. Statistical analyses were performed using the Mann-Whitney *U* test and a mixed effects model (Stata, College Station, TX).

**Results:**

Ninety-two percent of participants were women in their 40s. For SSc-RP patients, 69% had limited cutaneous SSc, the mean ± SD SSc duration was 11 ± 5 years, and 38% had prior digital ulcers—none currently. Baseline deoxyHb was higher, and O_2_ sat was lower, in SSc patients versus HC (*p* < 0.05). SSc patients had a greater decline in oxyHb and O_2_ sat from baseline to time 0 (after cold challenge) with distinct rewarming oxyHb, O_2_ sat, and deoxyHb trajectories versus HCs (*p* < 0.01). There were no significant correlations between oxyHb, deoxyHb, and O_2_ sat level changes following cold challenge and RCS or CHFS scores.

**Conclusion:**

Hyperspectral imaging is a feasible approach for SSc-RP quantification in the clinic setting. The RCS and CHFS values did not correlate with HSI parameters. Our data suggest that HSI technology for the assessment of SSc-RP at baseline and in response to cold provocation is a potential quantitative measure for SSc-RP severity and activity, though longitudinal studies that assess sensitivity to change are needed.

## Introduction

Raynaud phenomenon (RP) is a microcirculatory, vasospastic disorder characterized by visible color changes (blue = hypoxemia, white = ischemia, red = reactive hyperemia) most commonly in the fingers and toes in response to cold, emotional stress, or other environmental triggers. Raynaud phenomenon is classified as primary or secondary, with primary being idiopathic and secondary being related to an underlying disorder, especially autoimmune diseases including systemic sclerosis (SSc) [1–4]. Over 90% of SSc patients report RP, and the results of a UK study found that 46% of SSc patients report prior digital ulcerations during the course of their illness [5]. In a survey of 443 patients with self-reported primary and secondary RP, 64% reported poor ability to control attacks, and only 16% found at least one RP medication to be helpful [6]. While current evidence supports dihydropyridine calcium channel blockers or synthetic prostacyclin analogs as RP treatment, a recent systematic review highlighted the lack of evidence-based SSc-RP treatment [7]. Clinical trials designed to assess the efficacy of SSc-RP treatments are limited by a dearth of feasible and robust quantitative outcomes. The present study was undertaken to assess the feasibility and potential utility of hyperspectral imaging (HSI) as a quantitative outcome for SSc-RP randomized controlled trials (RCTs).

Herrick et al. discussed the difficulty of conducting RCT for SSc-RP given the disease heterogeneity, the effect of ambient temperature on RP activity, the impact of patients’ self-imposed lifestyle modifications, and the subjectivity of patient-reported outcomes [8]. The Raynaud’s Condition Score (RCS) and the appearance and/or healing of digital ulcers are commonly used validated patient-reported primary endpoints for SSc-RP RCTs [9]. The RCS rates the daily level of difficulty that a patient experiences directly due to RP from “no difficulty = 0” to “extreme difficulty = 11” on an 11-point Likert scale and scores are typically averaged over a 1- or 2-week period [10]. The Scleroderma Clinical Trials Consortium Vascular Working Group (SCTC-VWG) identified limitations for the RCS and RCS Diary that do not account for seasonal weather variations, patient efforts to ameliorate or avoid RP attacks, and habituation to RP symptoms [11]. Using the appearance of new, or the resolution of existing, digital ulcers as a trial endpoint is also problematic. The results of a web-based study where 50 rheumatologists from 15 countries were asked to grade a set of SSc digital lesions at two time points showed a lack of standardization amongst grading. While intra-rater reliability was high (0.81), inter-rater reliability was poor (0.46), suggesting that if digital ulceration is to be used as an SSc-RP trial outcome, standardized definitions are required [12]. Thus, identification and validation of quantitative SSc-RP outcomes and determination of their association with the patient experience are necessary to enable future valid SSc-RP clinical trial design.

Hyperspectral imaging (HSI) is a spectroscopic method that combines digital imaging with conventional spectroscopy and non-invasively measures oxygenated and deoxygenated hemoglobin (oxyHb and deoxyHb, respectively) and oxygen saturation (O_2_ sat) concentrations in the subpapillary skin plexus to a depth of 1–5 mm [13–15]. Oxyhemoglobin and deoxyHb values influenced by the microvascular volume of capillary density and Hb concentration are calculated to create an oxygenation map. An individualized reflectance or fluorescence spectrum is provided for each image pixel. Wavelengths of visual light in the 500–660-nm range, which includes oxyHb and deoxyHb absorption peaks, are collected from each image pixel and broken down by a spectral separator to generate a diffuse reflectance spectrum. These spectra are compared against standard transmission solutions to determine oxyHb and deoxyHb concentrations [14, 16–18]. Hyperspectral imaging has been shown to detect clinically relevant changes in skin microcirculation in patients with diabetes mellitus foot disease and those with peripheral artery disease (PAD) following revascularization surgery [13, 14]. Hyperspectral imaging technology may present a novel, fast, and effective method to quantify and monitor disease activity in SSc-RP. The aim of the current study is to assess the feasibility and potential utility of HSI to assess the microcirculation in patients with SSc-RP. To our knowledge, this is the first study to utilize HSI in patients with SSc-RP compared to HC.

## Methods

### Patient cohort

The Yale University Human Investigation Committee approved this prospective study (HIC# 2000026608). Patients who fulfilled the 2013 American College of Rheumatology SSc Classification Criteria with a history of RP were recruited from Yale Scleroderma Program outpatient clinics. Healthy control participants (HC) were recruited from the community [19]. SSc patients and HCs gave written informed consent for study participation in accordance with the Declaration of Helsinki. Healthy participants were excluded if they had RP and another rheumatic disease syndrome or were taking vasoactive medications or substances [20]. Study visits immediately following clinic appointments were conducted. Physical exam findings, disease subtype (diffuse or limited cutaneous SSc), SSc and RP disease duration, modified Rodnan skin score (mRSS), comorbidities, medications, and nailfold capillaroscopy (NFC) results obtained using a DermLite dermatoscope (3GEN Inc., San Juan Capistrano, CA) [21, 22] were recorded in a study-specific REDCap database.

### Hyperspectral imaging and cold challenge

Hyperspectral imaging was obtained using the hand-held HyperView™ camera (HyperMed, Waltham, MA). The camera probe generates oxygenation maps depicting oxyHb, deoxyHb, and O_2_ sat without physical patient contact.

In a temperature-controlled room (22 °C), participants placed their ring-free hands flat, side-by-side, palm side up, and atop a white sheet for 1 min to habituate the hands to room temperature. Baseline images of the palmar side of both hands were imaged, first at the center of the palm and then at the fingertips from right to left hand in sequential order for each time point. Subsequently, participants donned nitrile gloves to prevent evaporative cooling following cold water immersion [23] and immersed both hands in a cool water bath (15 °C [± 1 °C], measured by a calibrated thermometer in a standard container). After 1 min, participants removed their hands from the water bath and carefully removed the gloves with care not to wet their hands. The palmer hand surfaces were reimaged at 0 min, 10 min, and 20 min post-cold challenge.

**Fig. 1:**
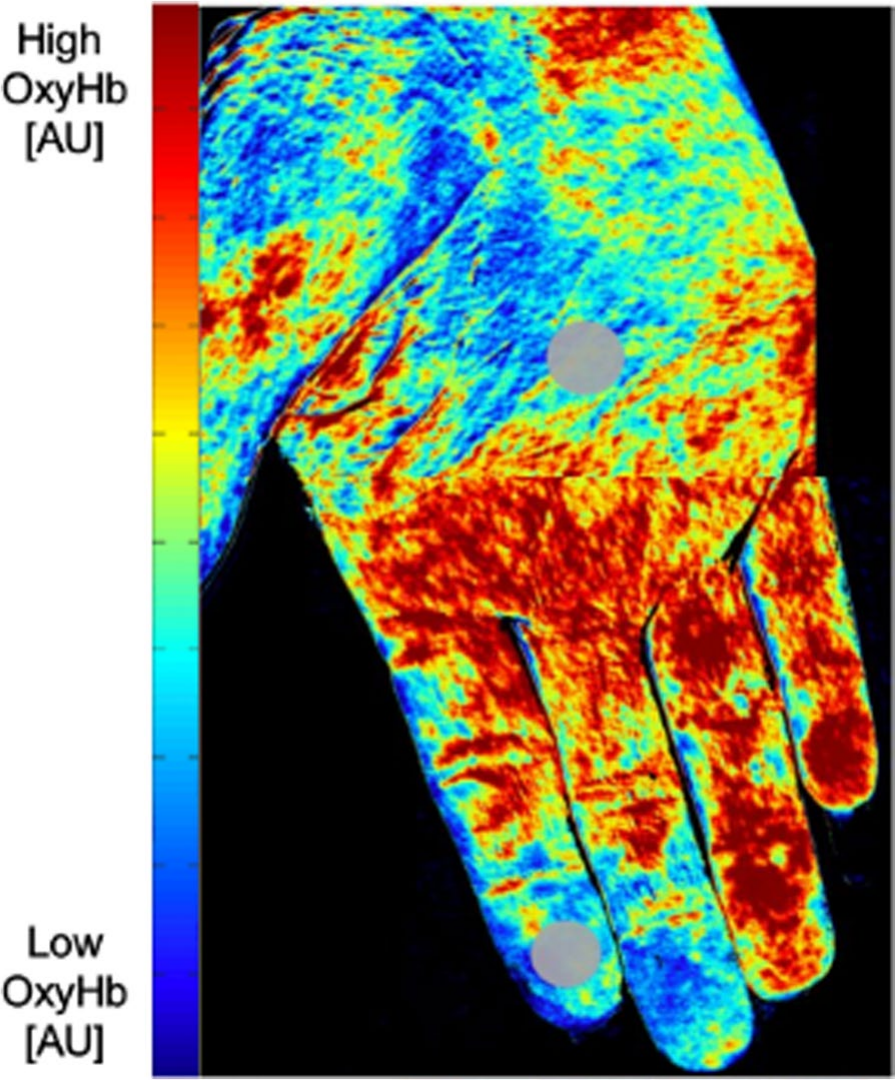
Hyperspectral imaging (HSI) heatmap. A HSI oxyHb image from a patient with systemic sclerosis-Raynaud phenomenon (SSc-RP). Red: higher oxyHb [arbitrary units (AU)]; blue: lower oxyHb (AU). The region of interest (ROI) on the palmar side of the hand and fingertips is indicated by an opaque gray circle

Using a default region of interest consisting of a circular area of 78 mm^2^ on the center of the palm and each fingertip (Fig. 1), oxyHb, deoxyHb, and O_2_ sat concentration data were generated using the OxyVu Hyperspectral Tissue Oxygenation Mapping System (HyperMed, Waltham, MA), as previously described [14, 17, 18]. The OxyVu-2™ probe generates a chemical composition gradient corresponding to a color spectrum viewed on a monitor. Blue arbitrarily represents low oxyHb, high deoxyHb, and low O_2_ sat while red denotes high oxyHb, low deoxyHb, and high O_2_ sat expressed in arbitrary units (AU).

### Patient-reported outcome (PRO) instruments

Patient-reported outcome questionnaires previously validated in SSc patients including the Cochin Hand Function Scale (CHFS) [24] and the Raynaud’s Condition Score (RCS) [9] were completed by each SSc patient participant following cold challenge. Briefly, the CHFS is a functional disability questionnaire comprising 18 questions about daily hand function-related activities (e.g., can you hold a bowl). The total score is obtained by adding the score of each question rated on a Likert scale from 0 (done without difficulty) to 5 (impossible to do) with a total score range of 0–90 [25]. Responses were manually entered into a study-specific REDCap database.

### Statistical analysis

Statistical analyses were conducted in STATA version 15.1 (College Station, TX) and presented using GraphPad Prism 7 (San Diego, CA). Between-group HSI differences were evaluated using the Mann-Whitney *U* test with statistical significance set at *p* < 0.05. A mixed effects linear model with an independent covariance structure was used to compare the slopes between individual HC and SSc participants from baseline to 0 min. A separate mixed effects model was applied to the rewarming phase post-cold challenge, in order to compare the slopes between individual HC and SSc participants from 0- to 20-min time points. To compare HSI parameters with PRO instruments, another mixed effects model was applied using the same covariance structure.

## Results

Twenty-five participants (13 SSc-RP and 12 HC) were enrolled (Table 1). The average age of SSc and HC participants was 49 and 40, respectively, and most were female (92% for both groups). SSc subjects had a mean and standard deviation (SD) SSc disease duration of 10.9 ± 5.2 years, and 69% of SSc subjects were classified as having limited cutaneous SSc (lcSSc). All patients self-reported RP symptoms [20], and 61% of patients were taking calcium channel blockers.

**Table 1:** Study participant characteristics

	SSc-RP, *n* = 13	HC, *n* = 12
Age (years), mean ± SD or as indicated	49.3 (± 19.9)	39.5 (± 11.9)
Sex (*n* (%), female)	12 (92.3)	11
SSc disease duration	10.9 ± 5.2	
SSc subtype (*n* (%))		
•LcSSc	9 (69.2)	
Modified Rodnan skin score	6.1 ± 4.5	
Nailfold capillaroscopy (*n* (%))		
•Early	1 (8)	
•Active	3 (23)	
•Late	3 (23)	
•N/A	6 (46)	
Serum autoantibodies (*n* (%))		
•ANA	10 (76.9)	
•Anticentromere	4 (30.8)	
•Anti-topoisomerase I	5 (38.5)	
•Anti-RNA polymerase III	1 (7.7)	
RP attack rate/2 weeks	31.5 ± 29.1	
Telangiectasias (*n* (%))	12 (92.3)	
Any clinically evident calcinosis cutis (*n* (%))	1 (7.7)	
Medications (*n* (%))		
•Calcium channel blocker	8 (61.5)	
•Aspirin	2 (15.4)	
•Statin	5 (38.5)	
•Mycophenolate mofetil	2 (15.4)	
•Mycophenolic acid	1 (7.7)	

*SSc-RP* systemic sclerosis-Raynaud phenomenon, *HC* healthy control, *ANA* antinuclear antibody, *SD* standard deviation

### Hyperspectral imaging at baseline and post-cold challenge

**Fig. 2:**
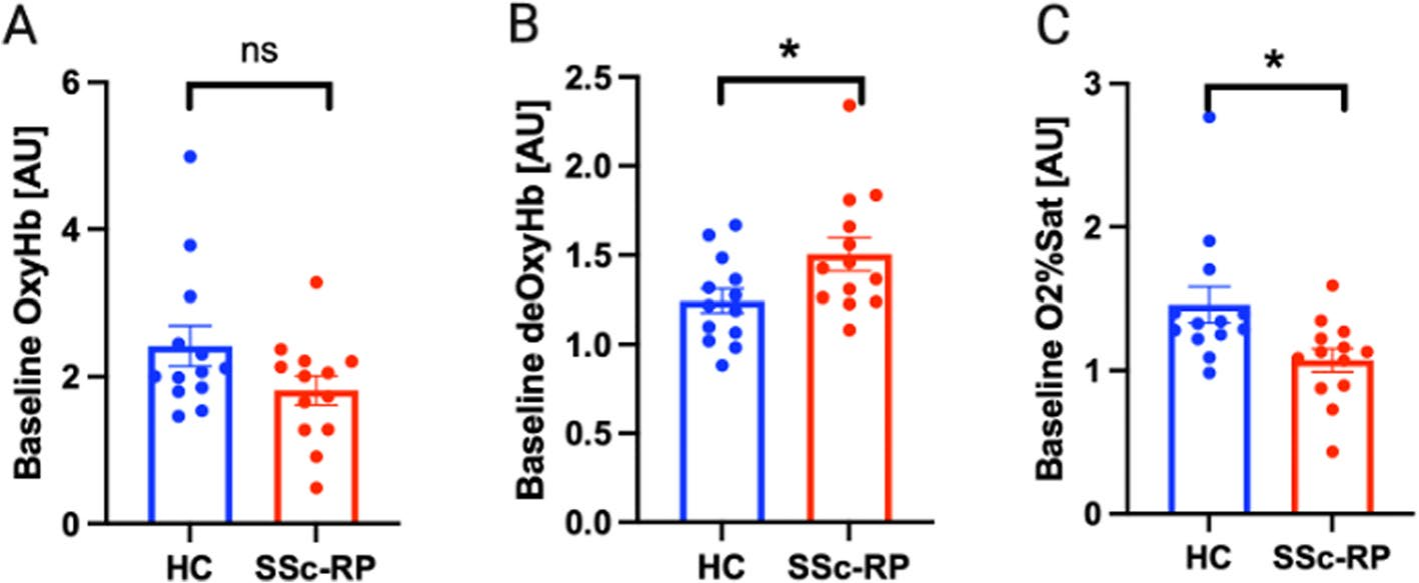
Baseline comparison of oxyhemoglobin, deoxyhemoglobin, and O_2_ saturation parameters in SSc-RP patients and HC. Prior to the cold challenge, there were no statistically significant differences noted at baseline in oxyHb between SSc-RP and healthy controls (HCs) (**A**), but SSc-RP had greater levels of deoxyHb (**B**) and lower levels of O_2_ sat compared to HC (**C**) (*p* < 0.05). No significance (ns), *p < 0.05, **p < 0.01

**Fig. 3:**
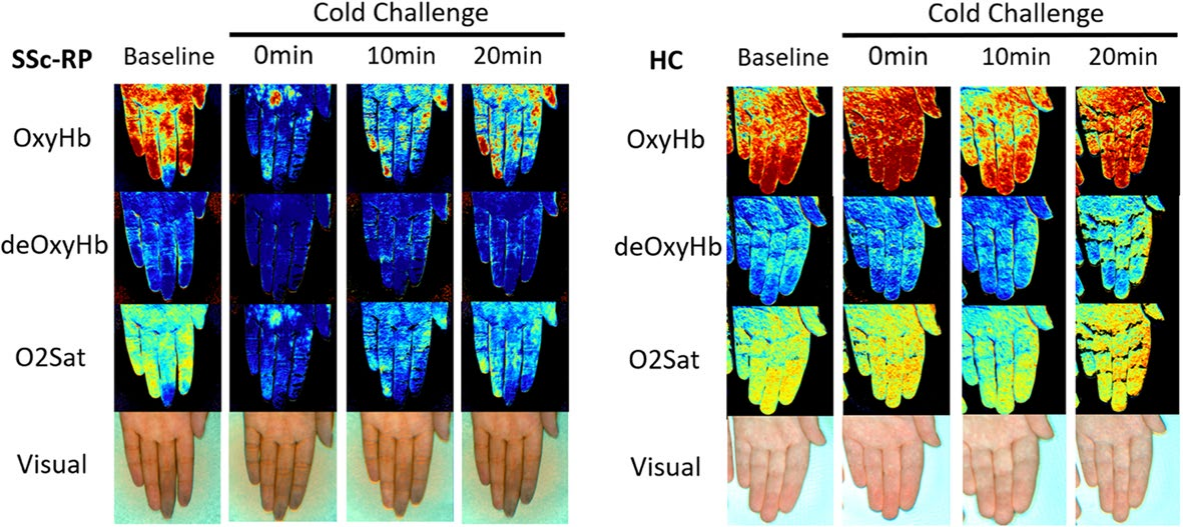
Representative images at baseline and 0, 10, and 20 min following cold challenge for SSc-RP and HC. HSI captures oxyHb and O_2_ sat gross visual changes in the fingertips during RP activity induced by cold water challenge. Hyperspectral images and the gross appearance of the hands were different between SSc-RP and HC with heatmaps for SSc-RP patients with more blue pixels for oxyHb and O_2_ sat, and more red pixels for deoxyHb, that correlates with visible fingertip pallor during RP activity induced by cold water challenge. Blue pixels for oxyHb and O_2_ sat and red pixels for deoxyHb were rare in HC images. Red: higher oxyHb [arbitrary units (AU)]; blue: lower oxyHb (AU)

Figure 2 demonstrates that at baseline, oxyHb levels were similar between SSc-RP and HC (mean 2.41 vs 1.86 AU, *p* = 0.22) (Fig. 2A); however, the SSc-RP group had higher deoxyHb compared to HCs (mean 1.24 vs 1.51 AU; *p* = 0.04) (Fig. 2B). Conversely, O_2_ sat was lower in the SSc-RP group compared to HCs (mean 1.46 vs 1.07 AU, *p* = 0.007) (Fig. 2C). Figure 3 demonstrates that hyperspectral images and the gross appearance of the hands were different between SSc-RP and HC with heatmaps for SSc-RP patients showing more blue pixels for oxyHb and O_2_ sat, and more red pixels for deoxyHb, that correlates with visible fingertip pallor. Blue pixels for oxyHb and O_2_ sat, and red pixels for deoxyHb, were notably rare in HC images.

**Fig. 4:**
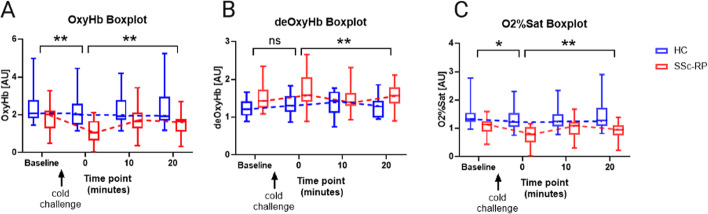
Comparison of oxyhemoglobin, deoxyhemoglobin, and O_2_ saturation parameters in SSc-RP patient and HC at baseline (prior to cold provocation) and post-cold provocation. After cold provocation, systemic sclerosis (SSc) patients had a threefold greater decline in oxyHb (**A**) and O_2_ sat (**C**) and minimal change in deoxyHb (**B**) from baseline (prior to cold provocation) to “time point 0” (immediately after cold provocation). Significant changes were observed in subsequent time points (10- and 20-min post-cold provocation) in oxyHb, O_2_ sat, and deoxyHb compared to HC. Mixed effects model. No significance (ns), *p < 0.05, **p < 0.01

SSc-RP subjects had a threefold greater decline in oxyHb and O_2_ sat images from baseline (prior to cold provocation) to “time point 0” (immediately after cold provocation) with significant changes in subsequent time points (10 and 20 min) in oxyHb, O_2_ sat, and deoxyHb compared to HC subjects (Fig. 4). The three variables changed minimally between baseline and “time point 0” in HCs. For oxyHb, the slope difference from baseline to “time point 0” in SSc-RP (slope − 0.71) vs HC (slope − 0.22) was − 0.48 (CI − 0.82, − 0.15; *p* = 0.005), with the SSc-RP group experiencing a greater decline in oxyHb following cold challenge. For deoxyHb, the slope difference from baseline to “time point 0” in SSc-RP (slope 0.18) vs HC (slope 0.10) was 0.07 (CI − 0.14, 0.29; *p* = 0.500), with SSc-RP groups experiencing a 1.8-fold increase in deoxyHb. Lastly, for O_2_ sat, the slope difference from baseline to “time point 0” in SSc-RP (slope − 0.33) vs HC (slope − 0.11) was − 0.22 (CI − 0.4, − 0.05; *p* = 0.014), with the SSc-RP group experiencing a threefold greater decline in O_2_ sat.

The slopes between “time point 0” (imaging immediately after cold challenge) and “time point 20” (imaging 20 min following cold challenge) were also calculated (Fig. 4). For oxyHb, the slope difference from “time point 0” to “time point 20” in SSc-RP (slope 0.60) vs HC (slope 0.07) was 0.53 (CI 0.22, 0.84; *p* = 0.001), with the SSc-RP group experiencing a greater increase in oxyHb. For deoxyHb, the slope difference from “time point 0” to “time point 20” in SSc-RP (slope − 0.21) vs HC (slope 0.02) was − 0.23 (CI − 0.38, − 0.08; *p* = 0.003), with the SSc-RP group experiencing a greater decline in deoxyHb. For O_2_ sat, the slope difference from “time point 0” to “time point 20” in SSc-RP (slope 0.29) vs HC (slope 0.02) was 0.26 (CI 0.13, 0.41; *p* < 0.001), with the SSc-RP group experiencing a greater increase in O_2_ sat.

### Association between oxyHb and patient-reported outcome (PRO) instruments

The association between CHFS scores and oxyHb at time 0 was − 0.032 (− 0.06, − 0.01) indicating the patients who reported worse hand function had lower mean finger oxyHb concentration (*p* < 0.05). The association between RCS scores and oxyHb at time 0 was − 0.005 (− 0.09, 0.08), indicating that patients who reported worse Raynaud symptoms had lower mean oxyHb concentration, but the result was not statistically significant.

## Discussion

RP is a microcirculatory vasospastic disorder reported by the majority of patients with SSc [3, 26, 27]. Even with treatments that include calcium channel blockers and vasodilatory agents, the condition can progress to digital gangrene with possible digital loss [28]. Herein, we demonstrate that HSI is a feasible approach for SSc-RP quantification in the clinic setting. We show that HSI at baseline and after cold challenge is a feasible and quantitative assessment to quantify SSc-RP disease.

To date, clinical trials in SSc-RP have been negative, which may be due to ineffective medications or the inadequacy of currently used RP outcome measures. Because insurance approval for RP treatments often is contingent upon regulatory agency approval, there is an unmet medical need to enhance the SSc-RP clinical trial design to enable valid testing of therapy. Herein, we demonstrated that HSI can be used in a clinic setting to non-invasively quantify SSc-RP hand microcirculation. We identified significant baseline differences in deoxyHb and O_2_ sat values between SSc-RP and HC subjects. Moreover, we observed differences in oxyHb and O_2_ sat values following cold challenge, as well as significantly different rewarming trajectories for oxyHb, deoxyHb, and O_2_ sat in patients with SSc (61% receiving calcium channel blocker therapy), as compared to HC. These findings suggest that the use of HSI is a feasible and potentially useful quantitative outcome for SSc-RP care and clinical trials, even in patients receiving vasodilation.

Cutaneous hand and finger microcirculation accommodates multiple functions, including nutrient supply and waste clearance as well as heat exchange [29]. Branches of the radial and ulnar arteries anastomose to form the superficial and deep palmar arches that give rise to the common and proper digital arteries. These arterial vessels branch to form the superficial subpapillary plexus that lies beneath the epidermal papillae, and the deep plexus that lies between the reticular dermis and the underlying intradermal fat. The superficial subpapillary plexus consists of a capillary loop for each dermal papilla and can be viewed with an ophthalmoscope or a dermatoscope [29, 30]. The primary role of these two plexi is nutritive blood flow. For skin thermoregulation, blood is shunted through arteriovenous anastomoses that are highly innervated by sympathetic nerves. In healthy individuals, exposure to cold or stress increases tone in arteriovenous anastomoses but does not impede nutritive capillary flow. Patients with secondary RP experience decreased flow in nutritive capillaries, thermoregulatory vessels, and upstream arterioles resulting in characteristic skin color changes and attendant ischemic complications [20, 31, 32].

At baseline, SSc patients displayed higher deoxyHb and lower O_2_ sat levels, but no difference in oxyHb levels vs HC participants, which may reflect the microvascular damage that occurs in patients with SSc-RP. Although the pathogenesis of SSc-RP vascular deformities is not fully understood, endothelial cell dysfunction [e.g., endothelial cell apoptosis, upregulation of adhesion molecules, and pericyte activation [31, 33–36], impaired red blood cell (RBC) deformability [37], imbalance of circulating vasodilatory and vasoconstrictor factors [38–41], increased circulating reactive oxygen species (ROS) [37], and increased basal and reactive sympathetic-mediated arterial tone [20, 42]] has been implicated. The result is digital artery intimal thickening and exaggerated cold and stress response [31]. One RP pathogenesis hypothesis is that chronic oxidative stress mediated by free radicals induces microvascular endothelial injury, resulting in tissue hypoxia with resultant inflammation and fibrosis [31, 43]. Giovannetti et al. showed increased circulating ROS in SSc patients compared to HC, which can cause oxidative stress and changes in red blood cell (RBC) structural homeostasis, such as cytoskeletal oxidative denaturation **[**37**]**. Loss of RBC structural integrity in SSc patients may alter adhesive properties and deformability, impairing RBC function. Furthermore, in a study by Konttinen et al., immunohistochemical analyses of SSc skin biopsies showed von Willebrand factor in the perivascular space and interstitial papillary dermal matrix, suggesting papillary dermal microvascular damage [43]. Additionally, functional vascular changes in SSc-RP patients include impaired endothelial-dependent vasodilation, possibly due to increased vasoconstrictor (endothelin-1) [38, 39] and decreased vasodilator (nitric oxide and prostacyclin) production, though variance in these molecules likely relates to disease activity and damage progression [40, 41]. Sympathetic tone alterations, important in skin thermoregulation, are also implicated in RP pathogenesis, namely by decreased vasodilation (e.g., calcitonin gene-related peptide (CGRP) deficiency) and increased vasoconstriction medicated by α2-adrenoreceptors [20, 42]. One potential reason may be that RP patients have a lower threshold for α2-adrenoreceptor activation than HC [31]. Such microvascular structural and functional changes and neuropathic alterations may account for baseline differences in the HSI oxygenation maps seen for deoxyHb and O_2_ sat in patients with SSc.

Our second important finding was that HSI quantitatively captured SSc patients’ exaggerated response to cold provocation challenge (baseline to “time point 0”) in oxyHb and O_2_ sat (even though 61% were receiving calcium channel blockade), but not in deoxyHb, levels compared to HC. Little change in slope between baseline (prior to cold challenge) and “time point 0” (imaging immediately after cold challenge) was noted in HC, possibly reflecting the ability of HCs to shunt blood through digital arteriovenous anastomoses to prevent heat loss (oxyHb = 0.07, deoxyHb = 0.02, O_2_ sat = 0.02). Conversely, SSc-RP patients showed greater absolute value baseline to “time point 0” slopes (oxyHb = − 0.71, deoxyHb = 0.18, O_2_ sat = − 0.33). Levels of oxyHb and O_2_ sat declined while deoxyHb levels increased compared to HC showing an abnormal response to cold provocation and the ability of HSI for quantification. Furthermore, we found the use of cold provocation elicited a more impressive change between HC and SSc-RP (Fig. 4) as compared to baseline imaging (Fig. 2), which may be useful for assessing change in response to treatment beyond calcium channel blockade.

The third potentially impactful finding was the significantly different rewarming trajectories for SSc-RP vs HC in oxyHb, O_2_ sat, and deoxyHb. In the HC group, minimal slope changes between “time point 0” (imaging immediately after cold provocation) and “time point 20” (imaging 20 min after cold challenge) were observed (oxyHb = 0.07, deoxyHb = 0.02, O_2_ sat = 0.02), as HC maintained tissue oxygenation post-cold challenge; however, SSc-RP patients showed greater baseline to “time point 0” slopes (oxyHb = 0.60, deoxyHb = − 0.21, O_2_ sat = 0.29) that indicated prolonged rewarming times. Patients with SSc-RP have decreased ability to properly shunt blood through dermal AV anastomoses due to exaggerated vasoconstriction of the digital arteries and cutaneous arterioles in response to cold or stress [44, 45].

Given the influence of external factors on PRO instruments in SSc-RP, research to identify more quantitative and objective measures is being conducted [7, 23, 46, 47]. Non-invasive imaging techniques that have been previously used to quantify SSc-RP severity and activity include nailfold capillaroscopy (NFC), thermography, and laser speckle contrast imaging (LSCI) methods. Nailfold capillaroscopy abnormalities detected using a dermatoscope or ophthalmoscope [48] are included in the American College of Rheumatology (ACR) 2013 SSc classification criteria underscoring the importance of microvasculature disease in SSc pathogenesis [49]. The NFC technique allows visualization of cutaneous capillaries (branches of the superficial subpapillary plexus) at the nailbed and identification of SSc-associated structural changes. However, the reliability of this technique remains an issue as nailfold capillaries cannot always be well visualized, especially in individuals with darker skin or those wearing dark nail polish, and capillary abnormality severity ratings are prone to scorer subjectivity [47, 50]. Thermography uses infrared emissivity to quantify surface temperature [°C] and has been used in SSc-RP clinical trials [40, 51, 52]; however, measurements are temperature-based and thus easily subject to spurious fluctuations. Laser speckle contrast imaging quantifies the movement of blood [values reported in arbitrary flux units (AU)] in microcirculation by directing a laser beam with a penetration depth of 100 to 500 μm to generate a speckle pattern [15, 23, 53]. Limitations of LSCI are that it is highly sensitive to hand movements that alter the angle at which the image is captured, is sensitive to ambient light, and its clinical relevance is unknown [15, 53–55]. Perhaps the best course of action to improve SSc-RP clinical trial design is to include multiple quantitative outcomes including thermography, LSCI, and HSI as sensitivity to clinically relevant changes remains unproven [56].

We did not find a robust correlation between the CHFS and RCS PRO measures and HSI parameters. Other groups have reported a lack of agreement between non-invasive measurements of digital perfusion for RP (e.g., thermography and LSCI) and existing PRO instruments [23, 46, 53]. In a trial evaluating the combination of aspirin and dipyridamole, patients reported RP symptom improvement that were discordant with a lack of change in objective digital perfusion techniques (LSCI and thermography) [57]. Reasons for this include the complex and dynamic nature of RP and differences in disease stages between patients (e.g., early disease, peak symptoms, resolution, scarring/fibrosis) and the patient response to the disorder such as catastrophizing or drastic changes in lifestyle to avoid RP attacks. It is likely that both non-invasive imaging modalities and PROs capture subtleties in the RP disease process and the patient responses [53, 58]. The newly developed and validated *Assessment of Systemic Sclerosis-Associated Raynaud’s Phenomenon (ASRAP)* patient-reported outcome instrument [59] addresses many of these shortcomings and will likely improve our ability to measure the change in SSc-RP patient symptoms.

Study strengths include our prospective study design within a well-characterized cohort of SSc subjects that included dermatoscope nailfold capillaroscopy assessments (standard-of-care) in a subset of patients **[**22**]**. We were able to perform HSI and cold challenge following regularly scheduled clinic appointments. Moreover, we obtained images with sufficient resolution that were amenable to quantitative analyses after minimal camera use training demonstrating the feasibility of the approach. Observed differences in oxyHb, deoxyHb, and O_2_ sat rewarming trajectories for SSc-RP vs HCs indicate HSI sensitivity, lack of compliance with therapy, and/or inefficacy of current treatments. Study limitations include the small number of patients with various SSc duration on various RP medications (e.g., calcium channel and angiotensin receptor antagonists) that confounds our ability to gain insights into the impact of disease duration and medications on HSI. The cross-sectional study design precluded assessment of HSI change over time that would have provided insights into the effect of season/weather, disease progression, and treatment effects. Future work will include imaging in a larger number of patients with varying RP disease duration, severity/damage and activity, performance of pre- and during treatment longitudinal imaging, and inclusion of the recently validated ASRAP patient-reported outcome instrument [59], that is specific for SSc-RP, to assess change over time. Additionally, upper extremity angiography, wrist brachial indices, and/or digital peripheral artery tonometry (EndoPAT) [32] were not performed and may be important correlates.

HSI may potentially be used to assess oxyHb, deoxyHb, and O_2_ sat changes in SSc-RP patients with the administration of various treatment regimes such as IV epoprostenol.

To our knowledge, this is the first study to utilize HSI for finger and hand imaging in patients with secondary RP compared to HC. We show that HSI at baseline and after cold challenge is a feasible and quantitative assessment that is amenable to in-clinic performance. Significant differences at baseline for deoxyHb and O_2_ sat and post-cold challenge rewarming trajectories in oxyhb, deoxyHb, and O_2_ sat between SSc-RP and HC suggest that the HSI approach is sensitive to change induced by cold provocation. Together, our results suggest that HSI technology may be a feasible and quantitative approach to assess RP vascular dysfunction and may be a useful outcome in SSc-RP clinical care and trials.

## Data Availability

The datasets used and/or analyzed during the current study are available from the corresponding author upon reasonable request.

## Abbreviations

RP: Raynaud phenomenon
SSc: Systemic sclerosis
HSI: Hyperspectral imaging
oxyHb: Oxygenated hemoglobin
deoxyHb: Deoxygenated hemoglobin
O_2_ sat: Oxygen saturation
HC: Healthy control
PRO: Patient-reported outcome instruments
RCS: Raynaud Condition Score
CHFS: Cochin Hand Function Scale
RCT: Randomized controlled trial
PAD: Peripheral artery disease
mRSS: Modified Rodnan skin score
SCTC-VWG: Scleroderma Clinical Trials Consortium Vascular Working Group
SD: Standard deviation
AU: Arbitrary units
NFC: Nailfold capillaroscopy
LSCI: Laser speckle contrast imaging
ACR: American College of Rheumatology
